# Heroin Abuse Results in Shifted RNA Expression to Neurodegenerative Diseases and Attenuation of TNFα Signaling Pathway

**DOI:** 10.1038/s41598-018-27419-9

**Published:** 2018-06-18

**Authors:** Mei Zhu, Yu Xu, Huawei Wang, Zongwen Shen, Zhenrong Xie, Fengrong Chen, Yunhong Gao, Xin Chen, Ying Zhang, Qiang Wu, Xuejun Li, Juehua Yu, Huayou Luo, Kunhua Wang

**Affiliations:** 1 Yunnan Institute of Digestive Disease, the First Affiliated Hospital of Kunming Medical University, Kunming, 650032 Yunnan China; 2 Department of Gastrointestinal surgery, the First Affiliated Hospital of Kunming Medical University, Kunming, 650032 Yunnan China; 3 Kunming Engineering Technology Center of Diagnosis and Treatment of Digestive Diseases, Kunming, 650032 Yunnan China; 4 Department of reproduction and genetics, the First Affiliated Hospital of Kunming Medical University, Kunming, 650032 Yunnan China; 5 Yunnan Drug Enforcement Commission Office, Kunming, 650032 Yunnan China; 6 Yunnan Drug Enforcement Administration, Kunming, 650032 Yunnan China

## Abstract

Repeated administration of heroin results in the induction of physical dependence, which is characterized as a behavioral state of compulsive drug seeking and a high rate of relapse even after periods of abstinence. However, few studies have been dedicated to characterization of the long-term alterations in heroin-dependent patients (HDPs). Herein, we examined the peripheral blood from 810 HDPs versus 500 healthy controls (HCs) according to the inclusion criteria. Compared with the control group, significant decreases of albumin, triglyceride, and total cholesterol levels were identified in HDPs (P < 0.001) versus HCs coupled with an insignificant decrease in BMI. Meanwhile, RNA-sequencing analyses were performed on blood of 16 long-term HDPs and 25 HCs. The results showed that the TNFα signaling pathway and hematopoiesis related genes were inhibited in HDPs. We further compared the transcriptome data to those of SCA2 and posttraumatic stress disorder patients, identified neurodegenerative diseases related genes were commonly up-regulated in coupled with biological processes “vesicle transport”, “mitochondria” and “splicing”. Genes in the categories of “protein ubiquitination” were down-regulated indicating potential biochemical alterations shared by all three comparative to their controls. In summary, this is a leading study performing a series of through investigations and using delicate approaches. Results from this study would benefit the study of drug addiction overall and link long-term heroin abuse to neurodegenerative diseases.

## Introduction

Drug addiction is a chronic disease, in which addicted people compulsively look for and take drugs, even though they are aware of its harmful consequences^1^. Drug abuse and addiction are major public health concerns, which lead to increased violence, prostitution, drug-related sexually transmitted diseases (STD) and death^2^.

Heroin, also termed as diamorphine, is a highly addictive, illegal drug. Medically it can be used to relieve pain. However, repeated administration of heroin results in the induction of dependence on heroin. The heroin-dependent patients (HDPs) are often associated with increased incidence of infectious diseases, as well as co-occurring medical conditions, including malnutrition, constipation, syphilis, HIV, Hepatitis C Virus (HCV), cardiovascular diseases, stroke, and mental disorders (such as depression)^3–5^. It has been reported that heroin is able to influence a variety of immune functions, which can directly act on immune cells through opioid receptors, and result in decreased *in vitro* T cell proliferation and cytokine production, and decreased phagocytosis and chemotaxis of macrophages. Thus, the immune dysregulatory effects of morphine could be potentially caused by alterations to the innate immune system, which is the body’s first line of defense against pathogens. Changes in cytokine and chemokine production would also influence the body’s inflammatory response. In addition, although some of the infections are due to passing the pathogens among HDPs through shared needles and unprotected sexual behaviors, chronic heroin abuse and addiction may result in permanent neuronal impairment in HDPs. In animal models, increased cerebellar neuronal apoptosis was observed in heroin-addicted rats^6,7^, possibly by activating the c-Jun kinase signaling and related pathways^8,9^. Mice that exposed to heroin prenatally showed reduced exploration for objects in novel locations indicating alterations in pyramidal neurons^10^. In humans, HDPs showed impaired white matter in anterior and superior regions with damaged myelin^11^. Other studies have shown inhibition in neural progenitor cells^12^, dysregulated postsynaptic density and endocytic zone^13^ and increased density of nitric oxide synthase expressing neurons^14^ were associated with heroin abuse and addiction.

However, a majority of human studies are either based on live brain imaging or post mortem specimens looking for protein biomarkers for specific neurons^12^. Although tremendous efforts have been spent on interrogating the signaling pathway and genetic factors underlying the drug rewarding circuitry^4^, little is known about how long-term heroin intake would potentially affect the neuronal system of these live HDPs. Therefore, a noninvasive test was applied to HDPs, which gives more information for overall transcriptional alterations in PBMC, especially compared to patients with neurological disorders.

So far there was no comprehensive study on blood biochemical parameters and transcriptome profiles of a large number of HDPs. In this study, we attempted to make a comparison between these parameters from 1509 HDPs and 500 HCs. Moreover, we performed RNA-seq on PBMC from 16 HDPs versus 25 HCs within comparable age range (all live subjects). Decreased expressions of TNFα pathway associated genes were observed in HDPs comparing to HCs, indicating impaired immune system in HDPs. After that, we performed a meta-analysis with neurological disorders and identified that the blood transcriptome of HDP mimic that of neurological disorders such as SCA2 (Spinocerebellar ataxia type 2) and PTSD (Posttraumatic stress disorder). In conclusion, this study has provided a noninvasive molecular diagnosis for degenerated neural systems in HDPs, deepened the understanding of addict mindset and paved the way for optimizing early interventions in therapeutics for these chronic addictive diseases, which would eventually reduce the burden of relapse and personal care.

## Results

### Characteristics of Participants in Study and Biochemical Blood Parameters for Heroin Addicts

**Figure 1:**
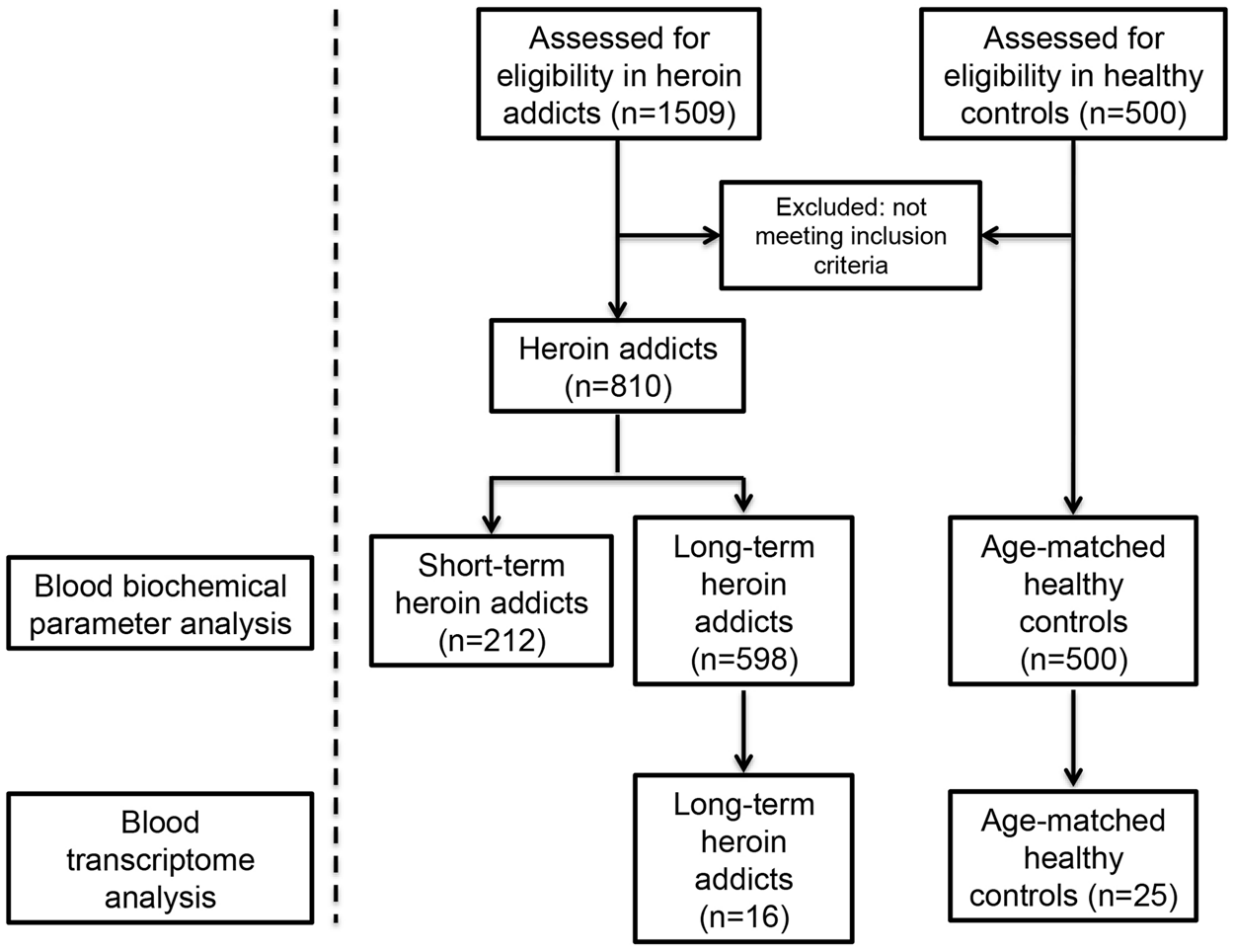
Overall experimental design for 2009 subjects including 1509 male HDPs and 500 male HCs in this study.

**Figure 2:**
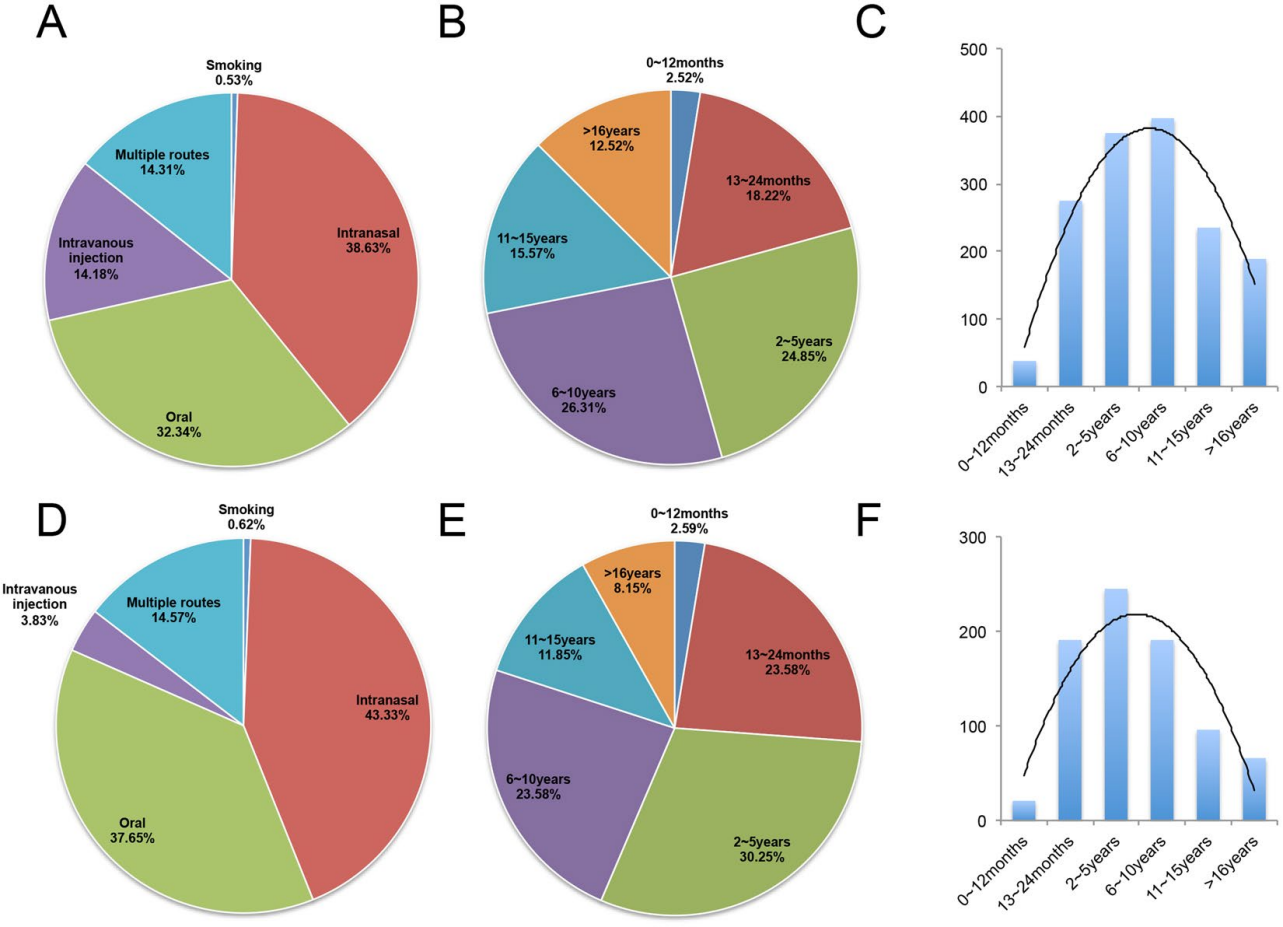
Characteristics of the routes of heroin administration and the average duration of heroin use in these HDPs. (**A**) The routes of heroin administration in all 1509 HDPs; (**B** and **C**) the percentage and actual number of HDPs with various duration of heroin use in all 1509 HDPs; (**D**) the routes of heroin administration in 810 enrolled HDPs; (**E** and **F**) the percentage and actual number of HDPs with various duration of heroin use in 810 enrolled HDPs.

Enrollment of participants occurred between July 2015 and December 2016. During this period, 2009 subjects were assessed for eligibility of this study including 1509 male HDPs and 500 male HCs within comparable age range (age ranging from 18 to 58, Fig. 1 and Table 1). The HDPs were registered at Kunming 5^th^ rehabilitation center and the history of heroin intake was recorded by questionnaire when admitted. The demographic data of 1509 HDPs and 500 HCs were summarized in Table 1 and the routes of heroin administration in these 1509 HDPs were shown in Fig. 2A. Overall, the routes of heroin administration were smoking (0.53%), intranasal (38.63%), oral (32.34%), intravenous injection (14.18%), and multiple routes (14.31%) (Fig. 2A). The average duration of heroin use was 93.3  ± 73.5 months (range 1~420 months, Table 1). Figure 2B,C summarized the duration heroin use in all HDPs in this study, which were 2.52% for HDPs less than 12 months, 18.22% for 13~24 months, 24.85% for 2~5 years, 26.13% for 6~10 years, 15.57% for 11~15 years, and 12.52% for more than 16 years (Fig. 2C). After an initial screening, 699 HDPs were excluded because they are positive for HIV (Human immunodeficiency virus), HBV (Hepatitis B virus), HCV (Hepatitis C virus) or Syphilis testing. A final sample of 810 male HDPs and 500 age- and gender- matched HCs were enrolled in the study. The demographic data of these 810 enrolled HDPs were summarized in Table 1. The routes of the heroin administration in these 810 HDPs were smoking (0.62%), intranasal (43.33%), oral (37.65%), intravenous injection (3.83%), and multiple routes (14.57%) (Fig. 2D). Comparing to the distribution of routes of heroin administration in all 1509 HDPs, a significant decrease in intravenous injection (14.18% to 3.83%) was observed in the distribution of routes of heroin administration in the 810 HDPs, suggesting a positive correlation of high risks of virus infections and the route of heroin administration via intravenous injection. The average duration of heroin use in these 810 HBPs was decreased to 77.3  ± 65.9 months (range 1~420 months, Table 1). Figure 2E, F summarized the duration of heroin use in these 810 HDPs of the final study, which were 2.59% for HDPs less than 12 months, 23.58% for 13~24 months, 30.25% for 2–5 years, 23.58% for 6~10 years, 11.85% for 11~15 years, and 8.15% for more than 16 years (Fig. 2F).

**Table 1:** Characteristics of healthy controls (HCs) and heroin-dependent patients (HDPs).

Study data	HCs	HDPs	p-value
**Preliminary study**			
No. of subjects	500	1509	NA
Sex	500 M/0 F	1509 M/0 F	NA
Age, y	36.0 ± 9.3 (20–58)	35.1 ± 8.1 (18–58)	0.734
BMI, kg/m^2^	25.3 ± 3.2	23.8 ± 3.8	0.232
Duration of heroin-use, mo	NA	93.3 ± 73.5 (1–420)	NA
**Main study**			
No. of subjects	500	810	NA
Sex	500 M/0 F	810 M/0 F	NA
Age, y	36.0 ± 9.3 (20–58)	33.9 ± 8.4 (18–56)	0.569
BMI, kg/m2	25.3 ± 3.2	23.6 ± 3.7	0.158
Duration of heroin-use, mo	NA	77.3 ± 65.9 (1–420)	NA

### Serum levels of Albumin, Triglyceride and total Cholesterol were altered in HDPs versus HCs

**Figure 3:**
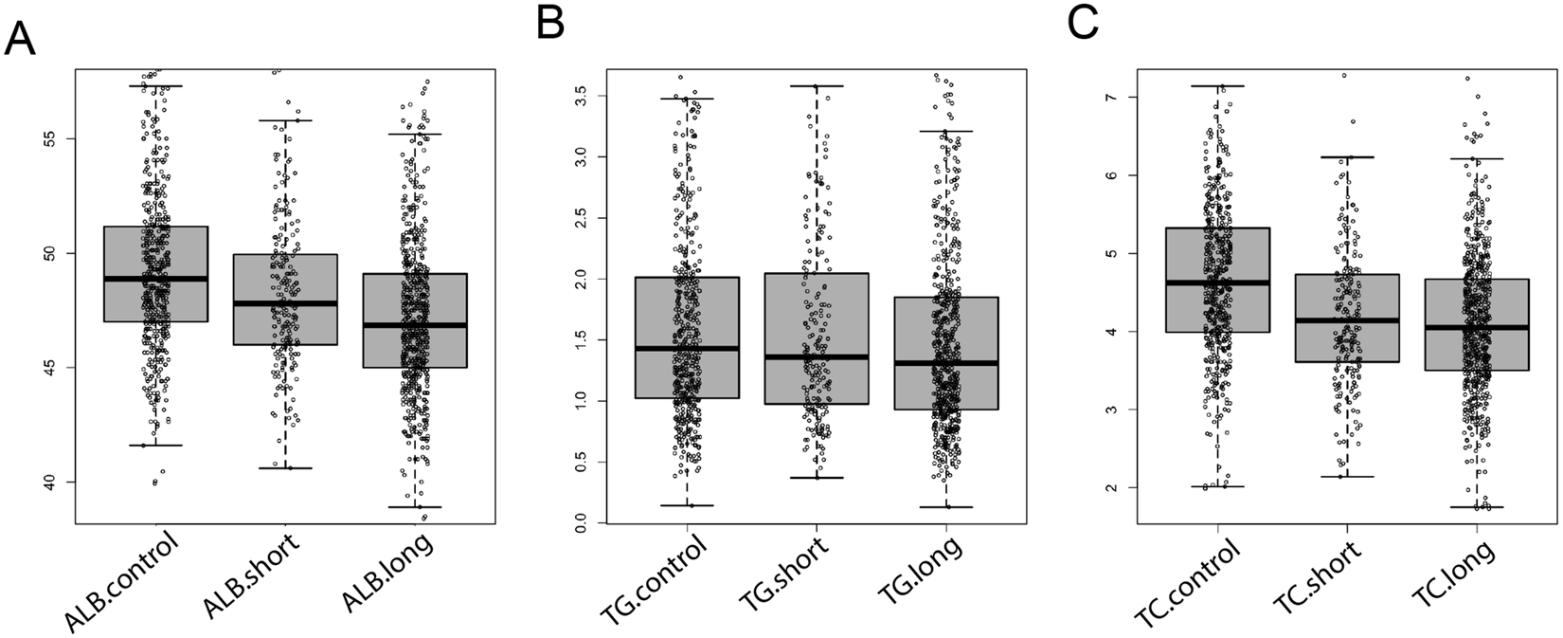
The albumin (Alb, **A**), triglyceride (Tg, **B**), and total cholesterol (Tc, **C**) levels were decreased with the elongation of heroin use, with short-HDPs significantly lower than HCs and long-HDPs significantly lower than short-HDPs.

To examine whether there is any difference in the peripheral blood parameters, we compared the data between HDPs and HCs for biochemical markers such as albumin (Alb), triglyceride (Tg), and total cholesterol (Tc) etc. According to the duration of heroin use (Fig. 2E and Table 2), we divided HDPs into short-term HDPs (short-HDPs, which are less than 24 months of addiction) and long-term HDPs (long-HDPs, which are more than 24 months of addiction). We identified that the Alb, Tg, and Tc levels were decreased with the elongation of heroin use, with short-HDPs significantly lower than HCs and long-HDPs significantly lower than short-HDPs (Fig. 3A–C). Low Alb levels often suggest impaired liver function, since albumin is secreted by hepatocytes and low Alb is often suggested with increased morbidity prediction after surgery^15–17^. This indicates that longer heroin use would gradually harm to the liver and potentially affect whole body function. These results are consistent with previous reports that Tg and Tc levels were significantly decreased in Methamphetamine users^18^, suggesting the HDPs are in a malnourished state. In addition, we also examined the BMI between HDPs and HCs. Consistent with previous findings in Methamphetamine addicts, HDPs showed lower average BMI than HCs, although it is not significant (Table 1). In general, drug users have lower BMI and lower percent fat mass than HCs despite similar or higher dietary intakes, and at similar levels of BMI, heavier drug use results in lower percentage body fat while weight loss is a significant predicting factor of morbidity^19–22^. All together, these results suggested that long-term heroin abuse leads to a gradual developing condition with malnutrition and predicts morbidity with weight loss and reduced Alb.

**Table 2:** Characteristics of healthy controls (HCs), long-term heroin-dependent patients (Long-HDPs) and short-term heroin-dependent patients (Short-HDPs).

Study data	HCs	Long-HDPs	Short-HDPs
No. of subjects	500	598	212
Sex	500 M/0 F	598 M/0 F	212 M/0 F
Age, y	36.0 ± 9.3 (20–58)	35.1 ± 8.3 (18–56)	30.5 ± 7.7 (18–56)
BMI, kg/m^2^	25.3 ± 3.2	23.2 ± 3.3	23.9 ± 3.9
Duration of Heroin-use, mo	NA	99.0 ± 64.6 (24–420)	17.4 ± 7.3 (1–24)

### Blood transcriptome of LT-HDPs cluster far away from HCs

Of the 810 HDPs and 500 HCs, 16 LT-HDPs (age ranging from 28 to 52) and 25 HCs within the comparable age range (age ranging from 32 to 48) were selected for whole transcriptome sequencing and analysis using PBMC samples from their whole peripheral bloods. The heroin usage ranges from 36 months to 86 months. The peripheral blood samples were collected according to proved ethical protocols for all 41 subjects and RNA-sequencing libraries were conducted after hemoglobin depletion. Overall, the 41 blood samples yielded over 180 G raw data from Illumina Hiseq4000 platform. On average, each sample produces ~20 million reads, ranging from ~18 million to ~25 million reads. Base quality and sequencing adapter trimming, reads mapping, biological replicates correlation analyses, and subsequent differential gene expression analyses were carried out using R 3.4.0 and Bioconductors. For base quality checking and sequence adapter trimming, quality limit 0.01, or the equivalent of Q20, was utilized to remove bases with a quality value of less than 20 as well as sequencing adapters. The filtered reads were then mapped to the Ensembl Human Genome reference genome (Accession number Ensembl GRCh38.79) via reference-guided assembly. Expression levels, which were calculated as fragments per kilo-base per million mapped reads (FPKM), were obtained for a total of 60,658 genes/transcripts annotated using Ensembl GRCh38.79.

**Figure 4:**
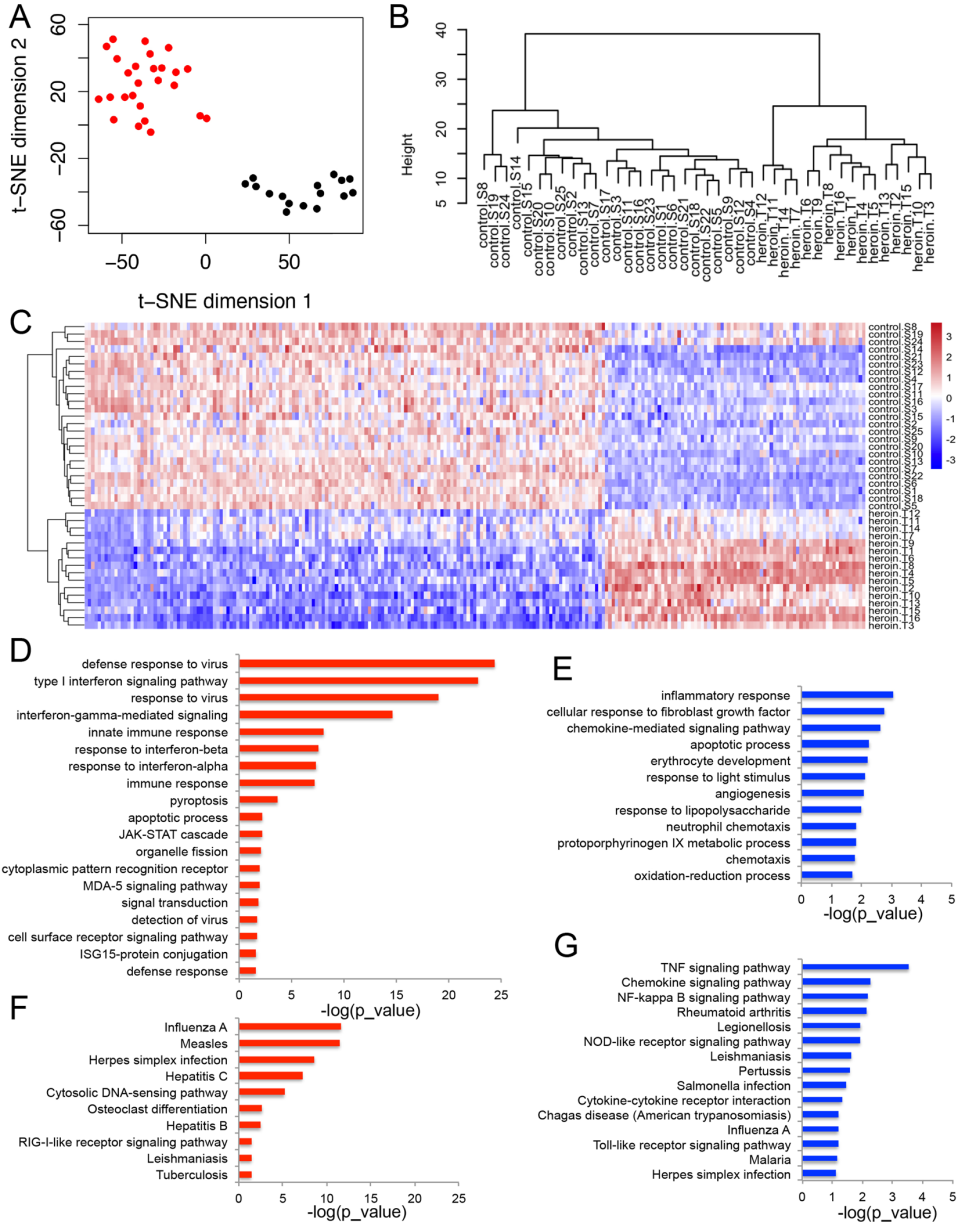
Transcriptome-sequencing analysis of 16 HDPs and 25 healthy controls. (**A**) The t-SNE projection showed that the whole transcriptome profiles of HDPs were distinct from those of HCs. (**B**) The unsupervised hierarchical clustering of whole transcriptome profiles showed that the group of HDPs were separated from HCs. (**C**) Top differentially expressed genes between HDPs and HCs were present in a heatmap. (**D**–**F**) DAVID Biological Process and KEGG pathway analyses of differentially expressed genes between HDPs and HCs.

A suite of biological replicates correlation analyses was firstly performed, including hierarchical clustering of samples and t-SNE (t-distribution Stochastic Neighbor Embedding) analysis. Subsequent analyses including hierarchical clustering of samples and sample-sample correlations analyses were performed using normalized FPKM values to determine the consistency of the gene expression profiles across samples. For hierarchical clustering of samples, Pearson correlation distance measure was employed together with average cluster linkage. In addition, we performed an unbiased clustering using t-SNE to observe the discrepancy in gene expression profiles between the HDPs and HCs (Fig. 4A). Similar to multidimensional scaling (MDS) or principle component analysis (PCA), t-SNE is a newly invented method to reduce dimensionality and preserve the distance between samples generating a 2D-map for visual inspection that accurately fits the general findings^23^. The 2D t-SNE projection showed that HDPs (black dots) and HCs (red dots) cluster far away at the diagonal corner of the plane. In line with the 2D t-SNE projection, hierarchical cluster of the samples also resulted in two separated clusters of HDPs and HCs, with smaller close clusters identified within these two top clusters respectively at an individual level (Fig. 4B). These results indicated that the global gene expression profiles in HDPs and HCs were distinctly different in peripheral blood samples.

To investigate the gene expression difference between HDPs and HCs, we performed one-way ANOVA test to pull out differentially expressed genes (Fold change >2, P < 0.01). In total there are 301 genes differentially expressed between HDPs and HCs. There are 193 genes out of 301 that were up regulated in HDPs and 108 were down regulated in HDPs compared with HCs (Fig. 4C).

### GO term analysis revealed up-regulated inflammatory response in HDPs

After obtaining the list of significantly differently expressed genes (DEGs), we performed GO term analysis by DAVID Bioinformatics Resources, to explore the potential biological and functional alterations in HDPs versus HCs. Firstly, we examined the MF (molecular function) analysis and found decreased gene expression in basic biochemical activities such as protein binding, chemokine activity, iron binding and so on and so forth (Fig. 4D, red). This suggests that the protein or iron modulated signaling induction is diminished in HDPs in general. To tear apart the functional correlation between these proteins, we then tested the DEGs in biological process (BP) and KEGG (Kyoto encyclopedia of genes and genomes). The top down regulated pathways (Fig. 4E, red) and top up-regulated pathways (Fig. 4F, blue) were represented with a cut-off (P < 0.05).

In BP analysis, we observed that compared to HCs, HDPs exhibited reduced gene expression involved in “inflammatory response”, “cellular response to FGF signaling stimulus” and “erythrocyte development” (the top 3 categories) and etc. These suggested that the inflammatory response pathway was suppressed. Looking into the genes in these categories, it includes the C-X-C subfamily of chemokines, which is critical for neutrophil attraction and activation (Fig. 2A): CXCL (C-X-C motif chemokine ligand) 1, CXCL5, CXCL8 and PPBP (Pro-Platelet Basic Protein); C-X-C ligand receptor: CXCR4 (C-X-C motif chemokine receptor 4); the NFκB inhibitors: NFKBIZ, NFKBID (NFKB inhibitor Zeta and Delta) and TNFAIP3 (TNF Alpha induced protein 3); other cytokines: IL1B (Interleukin 1 Beta) and THBS1 (Thrombospondin 1). The “cellular response to FGF signaling stimulus” category includes ZFP36 (Zinc finger protein 36), EGR3 (Early growth response 3), SNCA (Synuclein Alpha) and CXCL8. ZFP36 regulates mRNA stability of many important cytokines^24^ and sensitizes transformed cells lines to cell death^25^. EGR3 participates in lymphocyte development, endothelial growth and migration, and neuronal development^26^. It is known to regulate over 300 genes, of which 35% are involved in immune response cytokines^27^. The gene expression of “erythrocyte development” is impaired comparing to HCs, which indicates anemia associated with chronic heroin users is probably due to poor erythrocyte development and formation. In summary, the GO term BP revealed that HDPs have reduced expression of secreted chemokines and their receptors, inhibited TNFα pathways, and genes involved in neutrophil, lymphocyte and erythrocyte functions in contrast to HCs. The KEGG analysis also confirmed the results from BP, and we conclude that these chemokines would likely play a role in other signaling pathways (Fig. 2A).

### Meta-analysis of DEGs (versus corresponding controls) among HDPs, SCA2 and PTSD revealed significant overlap in up- or down- regulated genes respectively

**Figure 5:**
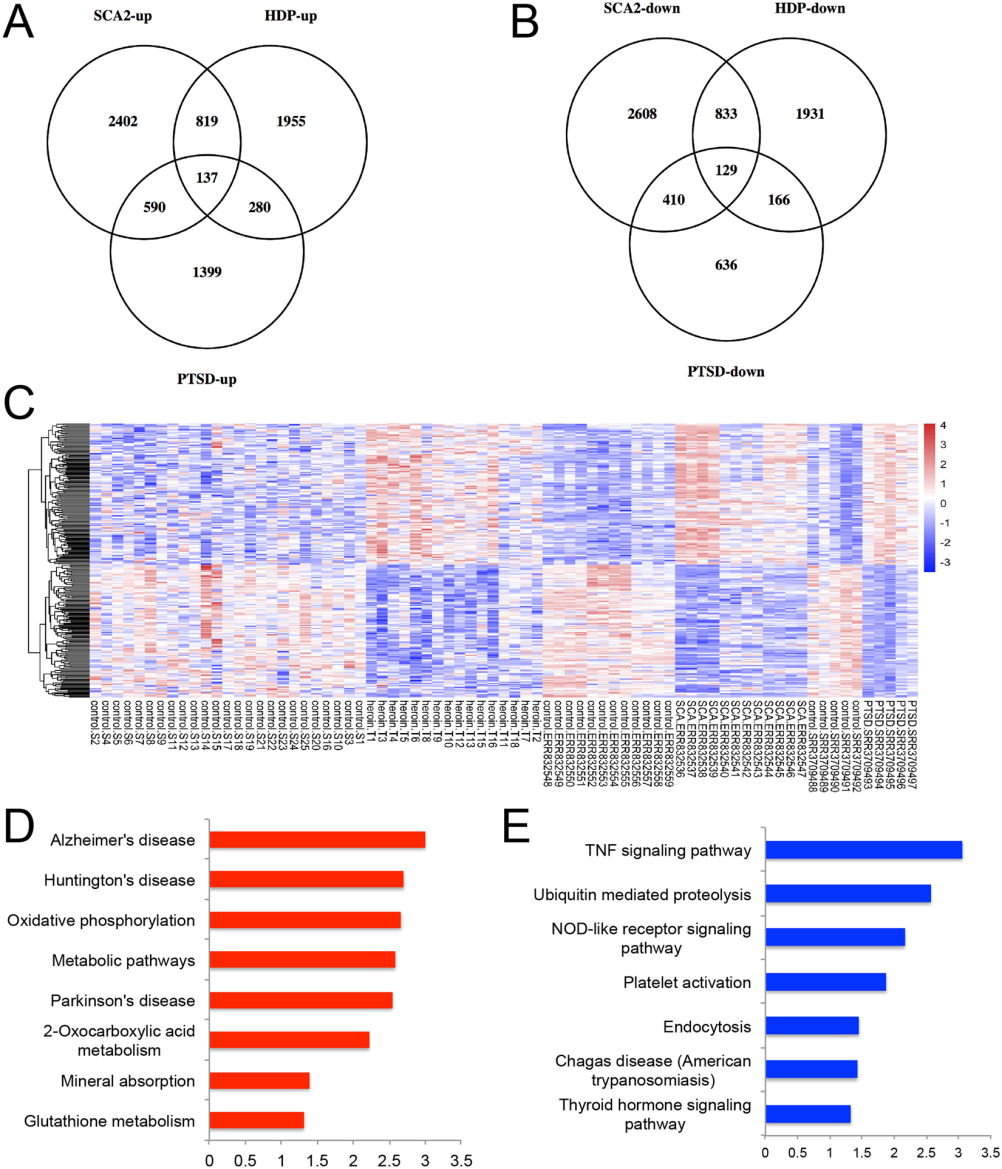
Transcriptome-sequencing analysis of HDPs, SCA2 and PTSD patients versus their healthy controls. (**A**–**B**) Venn diagram of overlapping differentially expressed genes in HDPs, SCA2 and PTSD patients versus their healthy controls. (**C**) Top differentially expressed genes in HDPs, SCA2 and PTSD patients versus their healthy controls were present in a heatmap. (**D**–**E**) DAVID Biological Process and KEGG pathway analyses of differentially expressed genes commonly identified in HDPs, SCA2 and PTSD patients versus their healthy controls.

Given that heroin dependent patients showed reduced white matter^11^ and damaged myelin in brain and unsanctioned opioid users^28^ showed perceived stigma, we hypothesized that HDPs may develop neurodegenerative phenotypes. Indeed, we observed that these HDPs often present inconsistency in memory recording and truth stating (Data not shown). We browsed existed studies about neurodegenerative diseases and performed meta-analysis of DEGs versus corresponding controls among HDPs, SCA2 (NCBI BioProject Accession: PRJEB8949 ID:291717) and PTSD peripheral blood mononuclear cell (PBMC) RNA-seq data^29^. Firstly we did analysis in each group, HDP, SCA2, and PTSD to obtain the DEG list, to exclude potential batch effects from different data resources. Then we compared among the groups of the DEGs. We identified that there are 130 overlapping genes in the up-regulated category (Fig. 5A,C) whereas about the same amount 129 common genes in the down-regulated category (Fig. 5B,C).

We further performed BP (Fig. 5D,E) and KEGG (Fig. 5F,G) pathway analysis for DAVID Bioinformatics Resources. In BP analysis, the up-regulated genes were enriched for cellular biochemical processes such as “vesicle-mediated transport”, “mitochondria”, and “splicing” etc. (Fig. 5D). This indicates that vesicle-mediated transport is enhanced in all three conditions, including DCTN1 (Dynactin 1), a protein binding both microtubules and cytoplasmic dynein. DCTN1 is reduced in mice with spinal and bulbar muscular atrophy (SBMA), a hereditary neurodegenerative disease and over-expression of DCTN1 mitigated neuronal toxicity of the pathogenic androgen receptor in a cell culture model of SBMA^30^. In the mitochondrial category, the enriched genes all belong to the NDUF family (NADH: Ubiquinone Oxidoreductase) containing subunit B11, A8, B7 and S8. Among these four, NDUFB11, NDUFA8 and NDUFB7 bear NADH dehydrogenase and oxidoreductase activities, whereas NDUFS8 is required for electron transfer and mutation in this gene is related with Leigh syndrome^31,32^. This indicates that HDPs, SCA2 and PTSD patients may have increased mitochondrial activities. Among the “splicing” group, the up-regulated genes include critical integral parts of the splicing machinery, such as SNPRB (Small Nuclear Ribonucleoprotein Polypeptides B and B1, SmB/B’)-core component of the SmB complex^33^, GEMIN7 (Gem Nuclear Organelle Associated Protein 7)-component of core SMN complex^34^, and SART1 (Squamous Cell Carcinoma Antigen Recognized By T-Cells 1)-essential for resembling tri-snRNP to the pre-spliceosome^35^. XAB2 is involved in splicing, DNA damage response (DDR) and transcription^36–39^, and related to the cockayne syndrome^40^, non-small cell lung cancer susceptibility^41^ and cancer stem cell maintenance^42^.

The BP of down-regulated genes revealed the top groups as “protein ubiquitination”, “small GTPase mediated signal transduction”, and “regulation of cytoskeleton” etc. (Fig. 5E). The top category “protein ubiquitination” genes mostly function in either E1, E2 or E3 complex of the ubiquitination process. Given that protein ubiquitination is necessary to degrade polyglutamine (polyQ) proteins in neurodegenerative diseases, reduced function of ubiquitination would result in accumulation of polyQ proteins in diseases such as SCA^43^. Failure to ubiquitinize and degrade certain mitochondrial proteins leads to neuronal diseases^44^. This indicates that in HDPs, SCA2 and PTSD, protein ubiquitination is attenuated, possibly resulting in accumulation of certain proteins causative for neuro-degeneration.

Next we interrogated the KEGG pathway and found the top up-regulated categories showed strong correlation with Alzheimer’s, Huntington’s and Parkinson’s diseases (Fig. 5F) by enriching genes such as ATP5D, ATP5H, HSD17B10 and NDUFB11, NDUFA8, NDUFB7, NDUFS8. These genes were also considered in the “Oxidative phosphorylation” and “Metabolic pathways”. In the down-regulated KEGG, we found inhibited “TNF signaling pathway” consistent with BP in HDPs versus HCs, indicating that HDPs, SCA2 and PTSD have abated TNFα signaling. Consistent with BP, “Ubiquitin mediated proteolysis” is also down-regulated in KEGG.

In summary, HDPs show high overlapping DEGs with SCA2 and PTSD respectively, two neural diseases with existing peripheral blood RNA-seq data sets. For common up or down regulated DEGs among all three groups, we identified classes of “vesicle-mediated transport”, “mitochondria”, “splicing”, and “Alzheimer’s, Huntington’s, Parkinson’s diseases” etc. to be up-regulated, while “protein ubiquitination”, “small GTPase mediated signal transduction”, “regulation of cytoskeleton” and “TNFα signaling” etc. to be down-regulated. These results suggested that HDPs share a lot in common with SCA2 and PTSD in terms of DEGs of PBMCs.

## Discussion

The biological impact of long-term heroin use is not well understood to live subjects. It is apparent that the global public health impact of this drug is quite significant. Clearly, the effects of *in vitro* experiments measure only direct heroin effect on cells, without taking into account indirect and secondary effects that propagate opioid-associated impacts within a biological system such as in the peripheral blood and the neural system.

Herein, we have provided a comprehensive characterization of the *in vivo* effects of heroin use in the peripheral blood of the HDPs. We have found that heroin brings down Alb, Tg, and Tc levels, as well as the BMI numbers, based on the time of addiction. Consistent with previous data of drug abuse including opioids, longer use of drug would result in malnutrition, loss of body fat, and increased morbidity. Our study has provided more data on a large numner of samples in heroin users to help understand the overall health states of drug addicts and aid in designing treating and nutrition supplements plans for drug abusers.

Our study is the first time to perform well-controlled RNA-sequencing analysis on PBMCs of HDPs versus HCs. We identified that HDPs exhibited reduced immune-response and cytokine secretion due to the down-regulated TNFα signaling pathways. This partially explains the gradual worsening malnutrition states of HDPs along time because of poor hematopoiesis and circulation. This also explains the phenomenon that heroin users are more susceptible to infections^45,46^. These findings provide a framework for understanding the effects of heroin across a highly relevant but complicated *in vivo* system and have identified signaling pathways of interest that may be playing key roles in immune-inhibitory actions of opioids.

More interestingly, given that chronic heroin dependents exhibit reduced white matter in the brain^11^, we were wondering whether there are molecular links between HDPs and neurodegenerative diseases. Based on the fact that SCA2 patients showed significant degeneration of white matter^47^ and PTSD patients presented many abnormalities in white matter integrity^48^, we downloaded RNA-seq data of PBMC from these two types of neurodegenerative diseases and performed the meta-analysis. Interestingly, we found that HDPs, SCA2 and PTSD share common up-regulated genes relative to independent corresponding controls in the category closely related to other neurodegenerative diseases such as Alzheimer’s, Huntington’s and Parkinson’s, of which Alzhermer’s disease patients also exhibited white matter degeneration in the brain^49^. This result shows that the molecular pathway underlying neural degenerative phenotypes of long-term HDPs may share a lot of similarities with common neurodegenerative diseases and studies towards these biochemical or functional pathways will hint the treatment of HDPs in terms of maintaining normal neural functions and then enable normal social recognition and interaction.

Another interesting finding of the common DEGs among the three groups include up-regulation of mitochondrial genes and splicing related genes. As we know that mitochondria is an important cell organelle regulating lots of biochemical and physiological functions. Splicing is critical to generate different functional transcripts of the same gene. It would be interesting to test changes of mitochondria proteins or their modifications to identify key candidates in modulating neurodegenerative diseases. Protein ubiquitination counts for a large portion of the common down-regulated genes, which suggests that looking for different ubiquitination profile in protein modification would be another access to pull the potential causing mechanisms common for HDPs, SCA2 and PTSD.

In conclusion this study for the first time identified the peripheral blood parameters using large amounts of well-controlled samples as well as performed RNA-seq for PBMC from HDPs and HCs. By analyzing the RNA-seq data, we identified important cellular signaling pathways were altered in HDPs versus HCs and HDPs share a majority of up or down DEGs with SCA2 and PTSD. This has paved way for more highlighted studies to investigate the indirect effects of drugs in the blood or nerve systems other than just on the cell *in vitro*.

## Materials and Methods

### Study Participants and Ethical Statement

The study participants were composed of 1509 male heroin addicts from Kunming 5^th^ rehabilitation center and 500 male healthy controls from the First Affiliated Hospital of Kunming Medicical University (Kunming, China), which were recruited in 2015 and 2016, with age ranging from 18~58 (Table 1), with social characteristics and drug-dependence history (duration of MA use, routes of drug administration, and daily dose were collected). Heroin abusers were patients in Kunming 5^th^ rehabilitation center before detoxification treatment. They were examined and identified as HIV negative and without active infections, inflammatory diseases or chronic systemic diseases. Additional drug tests were performed on urine samples of these patients, and those who exhibited other substance-positive were excluded from this study. Ethical approval was obtained from Clinical Research Ethics Committee, the First Affiliated Hospital of Kunming Medicical University. Methods indicating the study were approved by the First Affiliated Hospital of Kunming Medicical University and all methods were performed under an approved Institutional Review Board protocol. All methods were performed in accordance with the relevant guidelines and regulations. Prior to participation, the experimental procedures were explained to all the participants who gave their voluntary written informed consent. As indicated in our previous report that drug addicts tend to have a habit of drinking and smoking. In this study, we chose to enroll subjects who are smoking but with only occasional drinking behavior in both experimental and control groups.

### Blood sample collection and preparation

After overnight fasting (14~16 h), total of 6 ml blood samples were drawn from each subject, from which 2 ml were collected into EDTA-K2 tubes for hematological analysis, 2 ml were collected in plastic tubes without anti-coagulants for biochemical analysis, and the last 2 ml were collected with Trizol for down-stream molecular analysis.

### Hematological and biochemical analysis

Hematological analysis was performed using a Sysmex XT-2000iv automated hematology analyzer (Sysmex Corporation, Kobe, Japan). Biochemical analysis was carried out using an Architect c8000 analyzer (Abbott Laboratories, Abbott Park, USA). Hematological and biochemical parameters are listed in Table 2.

### RNA extraction and library construction

Among all 1509 subjects in this study, 16 long-term heroin addicts and 25 healthy controls with comparable age were selected for further transcriptome data analysis (Table 2). Intravenously collected peripheral blood samples were stored with Trizol XL (Amibon, USA) at −80 °C until down stream molecular analysis. Total RNAs were exacted using Qiagen miRNeasy Kit (Qiagen, USA) following the manufacturer’s protocols. For RNA-Seq library construction, globin transcripts were firstly depleted from total RNAs using the GLOBINclear kit (Life Technologies, USA) following the manufacturer’s protocols. All sequenced libraries were prepared using the TruSeq RNA Library Prep Kit v2 (Illumina, USA). All the libraries were sequenced on Illumina Hiseq4000 with 150 pb × 2 (PE). For population RNA-seq, ~20 M reads were generated (Pair-end 150 bp).

### RNA-Seq bioinformatics and statistics

Fastqc was used to perform quality control to all sequenced data. Data were trimmed by Trimmomatic to remove and filter low quality and adapter contaminated reads. We used the human genome NCBI GRCh38 and its corresponding transcriptome gene annotation for reads alignment. Tophat alignment tool (version 2.0.12) was used for alignment with default parameter settings. All data, including hematological and biochemical references, were presented as means ± standard errors. The two-way unbalanced analysis of variance (ANOVA) was used to examine the effect of sex, age, and sex-age interaction.

### Functional enrichment analysis annotation

Classifications of genes into biological process, molecular function, and cellular component GO categories were visualized with DAVID (Database for Annotation, Visualization and Integration Discovery Bioinformatics Resources version 6.8 (http://david.abcc.ncifcrf.gov). For KEGG pathway categorizations, DAVID Bioinformatics Resources version 6.8 was utilized with the Homo sapiens as the background organism. Threshold score was set at 0.05 when determining significantly enriched pathway categories.

### Data deposition

RNA-Seq data in this publication have been deposited in SRA under accession numbers PRJNA416408. All data generated or analyzed during this study are included in this published article or are available from the corresponding author on reasonable request.
